# Nuclear hBD-1 accumulation in malignant salivary gland tumours

**DOI:** 10.1186/1471-2407-8-290

**Published:** 2008-10-07

**Authors:** M Wenghoefer, A Pantelis, H Dommisch, W Götz, R Reich, S Bergé, M Martini, JP Allam, S Jepsen, S Merkelbach-Bruse, HP Fischer, N Novak, J Winter

**Affiliations:** 1 Department of Oral & Maxillofacial Plastic Surgery, University of Bonn, Sigmund-Freud-Str. 25, 53105 Bonn, Germany; 2 Department of Otorhinolaryngology/Surgery, University of Bonn, Sigmund-Freud-Str. 25, 53105 Bonn, Germany; 3 Department of Periodontology, Operative and Preventive Dentistry, University of Bonn, Welschnonnenstr. 17, 53111 Bonn, Germany; 4 Department of Oral & Cranio-Maxillofacial Surgery, Radboud University, Nijmegen, Geert Grooteplein-Zuid 14, 6525 GA Nijmegen, The Netherlands; 5 Department of Dermatology, University of Bonn, Sigmund-Freud-Str. 25, 53105 Bonn, Germany; 6 Department of Pathology, University of Bonn, Sigmund-Freud-Str. 25, 53105 Bonn, Germany

## Abstract

**Background:**

Whereas the antimicrobial peptides hBD-2 and -3 are related to inflammation, the constitutively expressed hBD-1 might function as 8p tumour suppressor gene and thus play a key role in control of transcription and induction of apoptosis in malignant epithelial tumours. Therefore this study was conducted to characterise proteins involved in cell cycle control and host defence in different benign and malignant salivary gland tumours in comparison with healthy salivary gland tissue.

**Methods:**

21 paraffin-embedded tissue samples of benign (n = 7), and malignant (n = 7) salivary gland tumours as well as healthy (n = 7) salivary glands were examined immunohistochemically for the expression of p53, bcl-2, and hBD-1, -2, -3.

**Results:**

HBD-1 was distributed in the cytoplasm of healthy salivary glands and benign salivary gland tumours but seems to migrate into the nucleus of malignant salivary gland tumours. Pleomorphic adenomas showed cytoplasmic as well as weak nuclear hBD-1 staining.

**Conclusion:**

HBD-1, 2 and 3 are traceable in healthy salivary gland tissue as well as in benign and malignant salivary gland tumours. As hBD-1 is shifted from the cytoplasm to the nucleus in malignant salivary gland tumours, we hypothesize that it might play a role in the oncogenesis of these tumours. In pleomorphic adenomas hBD-1 might be connected to their biologic behaviour of recurrence and malignant transformation.

## Background

The majority of salivary gland tumours are benign. Malignant salivary gland tumours are less frequent but occur in 15 to 32 percent of all patients. The most common point of origin for salivary gland tumours is the parotic gland in up to 80 percent of all cases [1].

Salivary gland tumours show a variety of different morphologic features which complicate the exact histomorphological diagnosis. They might be subdivided into salivary gland tumours with a) myoepithelial components, b) basaloid components, c) epithelial components, d) lymphatic components and e) pleomorphic adenomas [2].

In the investigation of the development of salivary gland tumours as well as in the prediction of their clinical course and possible treatment options, molecular biology has moved into the centre of tumour research. In this context proliferation associated antigens as Ki-67, proto-oncogenes as bcl-2, tumour suppressor genes as p53 or p21 and the overexpression of growth-factor binding receptors as HER-2 have been identified as important factors in the malignant progression of these tumours. [3].

Defensines as hBD-1, -2, -3 are positively charged peptides with molecular weights ranging from 3.5 to 6.5 kDa. Their ability to disintegrate membranes is responsible for their antimicrobial potency against a number of gram-positive and gram-negative bacteria as well as yeasts and certain viruses. Defensins have been detected in different tissues as the epithelia of the oral cavity, the gastrointestinal and respiratory tract, the urinary tract and the vagina as well as in salivary glands [4-7]. HBD-2 and -3 have been detected in the joint cartilage of patients with osteoarthritis thus being involved in the repair processes after cartilage damage in the cause of inflammation [8].

As defensins have been detected in the submandibular glands of patients with oral carcinomas [9] they might also play a role in tumour immunity and disintegration of tumour cells.

Whereas hBD-2 and -3 seem to be related to inflammation, the constitutively expressed hBD-1 might be involved in carcinogenesis. HBD-1 is located in a defensin gene cluster on the short arm of chromosome 8. There was a cancer specific loss of hBD-1 in 90% of renal clear cell carcinoma and 82% of malignant prostate cancer, whereas in benign epithelium the expression of hBD-1 was intact [10]. In oral squamous cell carcinomas of the oral cavity we found a 50-fold decreased, but not complete loss of human β-defensin-1 gene expression [11]. Therefore hBD-1 might function as 8p tumour suppressor gene and thus play a key role in control of transcription and induction of apoptosis in epithelial tumours [12].

This study was conducted to characterise proteins involved in cell cycle control and host defence in different benign and malignant salivary gland tumours in comparison with healthy salivary glands.

## Methods

In this study, 21 tissue samples of 7 patients with confirmed diagnosis of a benign, 7 patients with a malignant salivary gland tumour and 7 healthy salivary glands were investigated: 12 patients were of female and 9 patients were of male gender. The average age at diagnosis was 53.8 years. The healthy salivary gland tissue was collected from patients with head and neck tumours during the surgical procedure of neck dissection; those patients were neither irradiated nor received chemotherapy. Procedures involving the human tissue sampling collection followed a protocol approved by the ethical board of the University of Bonn.

After formalin fixation the tissue samples were embedded in paraffin and cut with a standard microtome (Reichert-Jung, Heidelberg, Germany) into 2 μm sections. Tissues for light microscopic evaluation were stained with Hematoxylin and Eosin (HE) and the diagnosis was confirmed by a pathologist (HPF). Immunohistochemistry was performed for p53, bcl-2, hBD-1, -2, and -3.

### Immunohistochemistry

Formalinfixed, paraffin-embedded tissue sections were used for immunohistochemical staining. After deparaffinization and dehydration, the slides were washed in Tris-buffered saline (TBS, Quartett GmbH, Berlin, Germany) containing 0.1% BSA. Endogenous peroxidase activity was quenched by incubating the slides in a solution of 700 μl H_2_O_2 _(30%) in 70 ml methanol. To perform antigen retrieval, the sections were pretreated with pepsin (0.4%) for 30 min at 37 °C. Blocking was done with normal serum. After incubation with primary antibody against p53, bcl-2, hBD-1, -2, and -3 (hBD-1 and p53: Biologo, Kronshagen, Germany; bcl-2: Quartett GmbH, Berlin, Germany; hBD-2: Santa Cruz Biotechnology, Heidelberg, Germany; hBD-3: Gentaur, Aachen, Germany) at room temperature for 60 min.

The slides were then washed in TBS buffer and the HRP-conjugated secondary antibody (DAKO, Hamburg, Germany) was added and the slides were incubated at room temperature for 30 min. Afterwards the slides were washed in TBS buffer and incubated with diamino benzidine tetrahydrochloride (DAB) as substrate and counterstained with hematoxylin (Merck Eurolab, Dietikon, Switzerland). Negative controls without primary antibody were included in each experiment to verify antibody specificity [13].

The immuno-staining for p53, bcl-2, and hBD-1, -2, and -3 was analysed using a Zeiss Axio-Imager A.1 microscope (Zeiss, Oberkochen, Germany). Tissue staining was assessed by using + (weak positive), ++ (positive), or - (negative).

To quantify the nuclear proportion, 100 cells per specimen were analysed for positive nuclear in comparison with cellular immunostaining in consecutive fields.

## Results

All tissue samples showed specific immunostaining. There was positive immunostaining for the defensins hBD-1, -2 and -3 in all tissue samples: In the benign salivary gland tumours (n = 7) and in the healthy salivary gland tissue (n = 7) hBD-1 was located in the cytoplasm. In the three pleomorphic adenomas there was additionally weak nuclear hBD-1 staining.

In the malignant tumours (n = 7) hBD-1 was located in the nucleus. In two of the malignant tumours there was additionally weak cytoplasmic hBD-1 staining. HBD-2 was detectable in the cytoplasm of the ducts in benign (n = 7) and malignant (n = 7) tumours as well as in the healthy tissue (n = 7). HBD-3 was located mostly in the cytoplasm of all tissues, but in one adenoid cystic carcinoma particularly in the nucleus as well.

P53 was positive in five of the malignant and two of the benign salivary gland tumours. The positive p53-immunostaining was very weak and occurred in two oncocytomas.

Bcl-2 was detected only in three of the malignant, but in six of the benign salivary gland tumours. There was no correlation between p53 and bcl-2 immunostaining.

All results are summarised in table 1 and 2.

**Table 1:** Location of hBD-1, -2, -3, p53 and bcl-2 in malignant, benign and healthy salivary gland tissues (positive ++, weak positive +, negative -)

**tumour**	**behaviour**	**hBD-1**	**hBD-2**	**hBD-3**	**bcl-2**	**p53**
**salivary duct carcinoma**	malignant	nucleus (and cytoplasm+)	cytoplasm++	cytoplasm ++	negative-	positive+
**mucoepidermoid carcinoma**	malignant	nucleus++ (and cytoplasm+)	cytoplasm++	cytoplasm++	positive++	positive+
**adenoid cystic carcinoma**	malignant	nucleus++	cytoplasm++	cytoplasm++ and nucleus+	negative-	positive+
**adenoid cystic carcinoma**	malignant	nucleus++	cytoplasm++	cytoplasm++	negative-	negative
**adenoid cystic carcinoma**	malignant	nucleus++	cytoplasm++	cytoplasm++	negative-	negative
**carcinosarcoma**	malignant	nucleus++	cytoplasm++	cytoplasm++	positive++	positive+
**adenocarcinoma**	malignant	nucleus++	cytoplasm++	cytoplasm++	positive++	positive+

**pleomorphic adenoma**	benign	cytoplasm++ (and nucleus+)	cytoplasm++	cytoplasm++	positive++	negative
**pleomorphic adenoma**	benign	cytoplasm++ (and nucleus+)	cytoplasm++	cytoplasm++	positive++	negative
**pleomorphic adenoma**	benign	cytoplasm++ (and nucleus+)	cytoplasm++	cytoplasm++	positive++	negative
**cystadenolymphoma**	benign	cytoplasm++	cytoplasm	cytoplasm++	negative-	negative
**cystadenolymphoma**	benign	cytoplasm++	cytoplasm	cytoplasm++	positive++	negative
**oncocytoma**	benign	cytoplasm++	cytoplasm	cytoplasm++	positive++	weak positive+
**oncocytoma**	benign	cytoplasm++	cytoplasm++	cytoplasm++	positive++	weak positive+

**healthy salvary gland tissue 1**	healthy	cytoplasm++	cytoplasm+	cytoplasm++	negative-	negative
**healthy salvary gland tissue 2**	healthy	cytoplasm++	cytoplasm++	cytoplasm++	negative-	negative
**healthy salvary gland tissue 3**	healthy	cytoplasm+	cytoplasm++	cytoplasm++	negative-	negative
**healthy salvary gland tissue 4**	healthy	cytoplasm++	cytoplasm+	cytoplasm++	negative-	negative
**healthy salvary gland tissue 5**	healthy	cytoplasm++	cytoplasm+	cytoplasm++	negative-	negative
**healthy salvary gland tissue 6**	healthy	cytoplasm++	cytoplasm++	cytoplasm+	negative-	negative
**healthy salvary gland tissue 7**	healthy	cytoplasm++	cytoplasm++	cytoplasm++	negative-	negative

**Table 2:** Cellular Localization of hBD-1, -2 and -3 in malignant salivary gland tumours in comparison with healthy salivary gland tissue

	**hBD-1**			**hBd-2**		**hBD-3**	
	Total Number of Cells	Nuclear Staining	%	Nuclear Staining	%	Nuclear Staining	%

Healthy	700	22	3	62	9	31	4

Malignant	700	525	75	88	13	302	43

**Figure 1:**
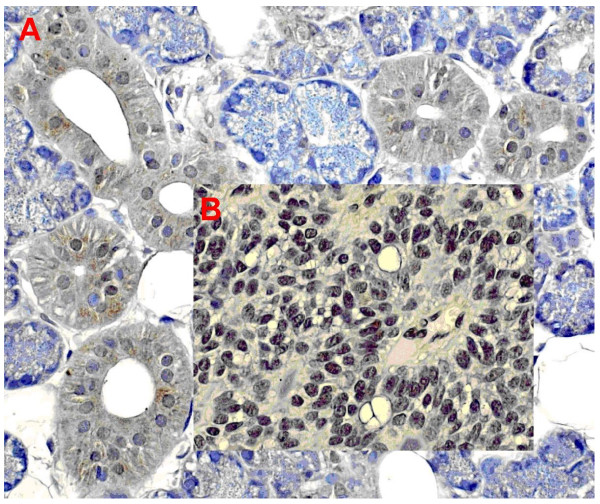
hBD 1 staining in healthy salivary gland tissue (A) and adenoid cystic carcinoma (B).

**Figure 2:**
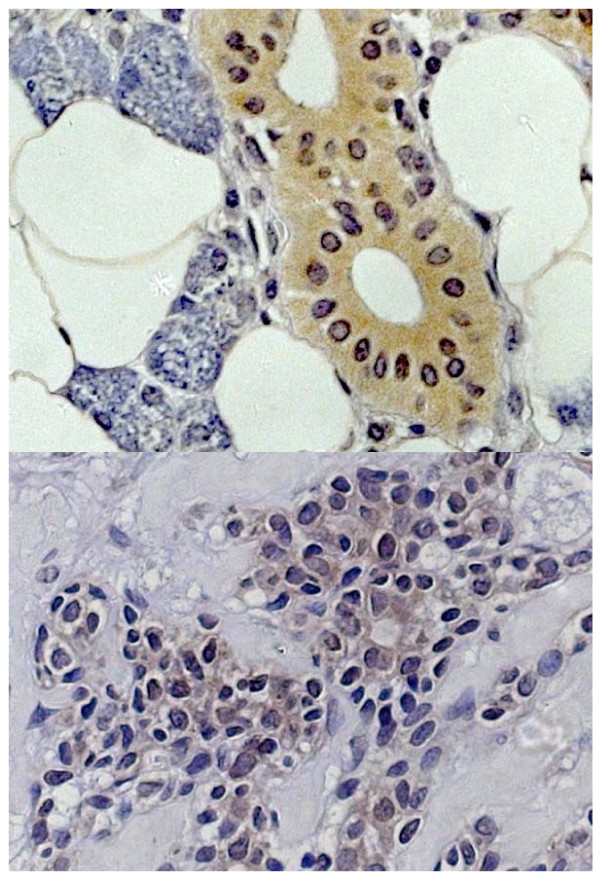
hBD-2 staining in healthy salivary gland tissue (above) and adenoid cystic carcinoma (below).

**Figure 3:**
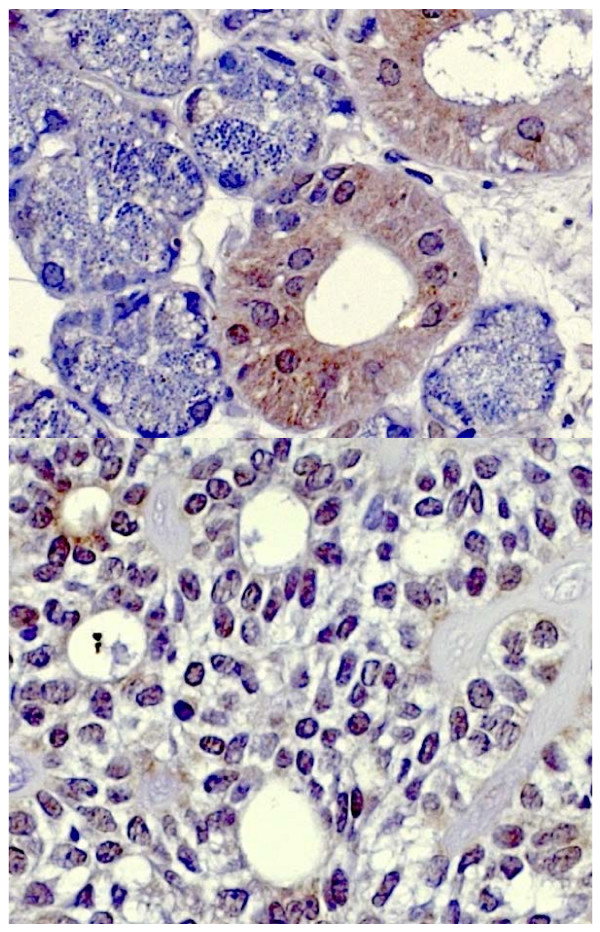
**hBD-3 staining in healthy salivary gland tissue (above) and adenoid cystic carcinoma (below)**.

**Figure 4:**
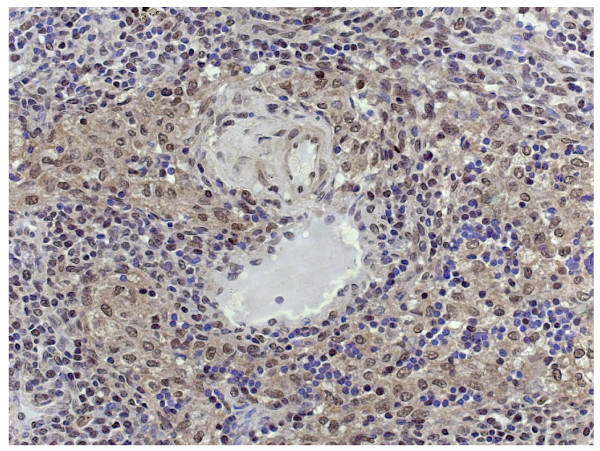
Nuclear and cytoplasmatic hBD-1 staining in a pleomorphic adenoma.

Fig. 1 shows the typical hBD-1 staining in an adenoid cystic carcinoma in comparison with healthy salivary gland tissue (fusion figure). Fig. 2 shows hBD-2 and Fig. 3 shows hBD-3 staining in adenoid cystic carcinomas (below) in comparison with healthy salivary gland tissue (above). Fig. 4 shows nuclear and cytoplasmatic hBD-1 staining in a pleomorphic adenoma.

## Discussion

In the present study the protein expression of human beta-defensins hBD-1, -2, -3, the tumour suppressor gene p53 and the proto-oncogene bcl-2 was analyzed in healthy salivary gland tissue in comparison with different benign and malignant salivary gland tumours. The most interesting finding indicated, that hBD-1 seems to be shifted from the cytoplasm to the nucleus of the tumour cells in malignant salivary gland tumours. Different benign and malignant tumours were chosen, to demonstrate that the translocation of hBD-1 was to be independent from the type of tumour.

HBD-1 is known to be constitutively expressed in epithelial tissues [6], whereas hBD-2 and -3 are inducible by inflammatory mediators as IL-1β. HBD-2 and -3 are connected to inflammation underlined by the works of other authors as for example Varoga and Paulsen who investigated both mediators in the cause of chronic inflammation of joint tissues as seen in osteoarthritis or rheumatoid arthritis [8,14,15].

There is evidence of a cancer specific loss of hBD-1 in 90% of renal clear cell carcinoma and 82% of malignant prostate cancer [10]. In squamous cell carcinomas of the oral cavity we found a 50-fold decreased gene expression of hBD-1 compared to healthy gingiva. In benign lesions of the oral mucosa as irritation fibromas or leukoplakias the hBD-1 gene expression was also decreased (5-fold in irritation fibroma and 2.5-fold in leukoplakia). These observations underline the hypothesis that hBD-1 might function as tumour suppressor gene and thus play a key role in the malignant progression of epithelial tumours [11]. Nevertheless the hBD-1 protein was detected mostly in the nucleus of the malignant salivary gland tumours. Therefore it is surprising, that the protein accumulates in the nucleus although the gene might be down regulated. Maybe by nuclear accumulation the hBD-1 protein down-regulates its own promoter. Promoter autoregulation is a well known effect and has been reported for other proteins before [16].

Loss of p53 tumour suppressor gene function is one of the most common genetic events described in human cancer [17]. Immunohistochemical staining for p53 usually shows mutant-type of p53, because the half-life of wild-type p53 protein is too short to detect. Wild-type p53 contributes to tumour suppression through at least two mechanisms: In response to DNA damage p53 causes the arrest of cell proliferation and/or the induction of apoptosis [18-20]. It has been suggested that p53 may induce apoptotic cell death by down-regulating bcl-2 and up-regulating bax expression [21,22].

The loss of p53 tumour suppressor function leading to deregulations in the apoptotic pathway might cause resistance of certain tumour cells to chemotherapy and radiotherapy [23].

In this study, an interrelation between hBD-1 and p53 as tumour suppressor or bcl-2 as proto-oncogene, was not evident. This might be, for different entities of salivary gland tumours were investigated to underline the effect of the hBD-1-translocation in malignant tumours versus healthy salivary gland tissue or benign tumours. P53 for instance is known to be undetectable in mucoepidermoid carcinomas [2]. The traceability of the presently known tumour-suppressor- or proto-onco-genes might be related to the tumour entity, whereas nuclear accumulation of the hBD-1 seems to be independent from the tumour entity, but related to malignancy.

Sherman and Froy reported that hBD-1 expression is up-regulated by c-myc expression, suggesting that c-myc regulates hBD-1 expression via a non-inflammatory pathway [24]. As c-myc is implicated in human cancers, and overexpression of c-myc at the protein and/or mRNA levels has been observed in virtually all types of cancers [25] a hBD-1 involving pathway might play a role in cancerogenesis.

There have been recent reports that the hBD-1 sequence shows homology with known cationic nuclear localized signal sequences in human keratinocytes. Furthermore nuclear localization of hBD-1 in human keratinocytes was described, which suggests a role for this peptide in gene expression [26]. The reason and biological impact of its nuclear translocation in malignant salivary gland tumours is not clear. Further studies should investigate whether hBD-1 protein accumulation is related to a down-regulation of hBD-1 gene expression as it was described in renal clear cell carcinoma or malignant prostate cancer [10]. We have the hypothesis that hBD-1 has abilities of a tumour suppressor gene. Nuclear accumulation of hBD-1 protein could be involved in the malignant progression of salivary gland tumours.

Pleomorphic adenomas are benign mixed tumours. They represent the most common neoplasms of the major salivary glands. It is not uncommon for pleomorphic adenomas to recur, and, though benign a subset will undergo malignant transformation [27]. As there was cytoplasmic as well as weak nuclear hBD-1 staining in the investigated pleomorphic adenomas in this study, this observation might underline the role of nuclear hBD-1 translocation in the ability of these tumours to recur or undergo a malignant transformation and should be observed in a greater subset of pleomorphic adenomas.

## Conclusion

1. HBD-1, -2 and -3 is traceable in benign as well as malignant tumours of the salivary glands.

2. We hypothesize that the nuclear shift of hBD-1 might be associated with malignancy in these tumours.

3. Pleomorphic adenomas show nuclear as well as cytoplasmatic hBD-1 staining which might be connected to their biologic behaviour of recurrence and malignant transformation.

## Competing interests

The authors declare that they have no competing interests.

## Authors' contributions

HPF confirmed the diagnosis of all tumours. SM-B carried out the immunohistochemistry. JW, AP and MW conceived of the study, and participated in its design and coordination. HD, WG and SJ participated in the design of the study. JPA and NN drafted the manuscript. RR, SB and MM performed the operations and coordinated the tissue sampling and preparation. All authors read and approved the final manuscript.

## Pre-publication history

The pre-publication history for this paper can be accessed here:


